# Assessment of Microfinance Interventions and Intimate Partner Violence: A Systematic Review and Meta-analysis

**DOI:** 10.1001/jamanetworkopen.2022.53552

**Published:** 2023-01-27

**Authors:** Lao-Tzu Allan-Blitz, Rose Olson, Quang Tran

**Affiliations:** 1 Division of Global Health Equity, Department of Medicine, Brigham and Women’s Hospital, Boston, Massachusetts; 2 Harvard Medical School, Boston, Massachusetts; 3 Cambridge Health Alliance, Cambridge, Massachusetts; 4 Department of Counselling, Developmental and Educational Psychology, Boston College, Chestnut Hill, Massachusetts

## Abstract

**Importance:**

An estimated 27% of ever-partnered women aged 15 to 49 years have experienced intimate partner violence (IPV) in their lifetimes, which has been associated with a wide range of both acute and chronic illness. Poverty is thought to be a major driver of IPV, and economic empowerment programs may reduce violence.

**Objective:**

To evaluate whether microfinance interventions are associated with reductions in various forms of IPV.

**Data Sources:**

On August 3, 2022, PubMed, CINAHL, Embase, Web of Science, EconLit, and 5 global health databases were searched from inception.

**Study Selection:**

Included studies were randomized clinical trials evaluating the effect of microfinance interventions vs control on exposure to IPV. This study was conducted following the Preferred Reporting Items for Systematic Reviews and Meta-analyses reporting guideline.

**Data Extraction and Synthesis:**

Authors independently assessed study eligibility, extracted prespecified data, and evaluated risk of bias using the Cochrane Risk of Bias tool.

**Main Outcomes and Measures:**

Outcome measures of interest were exposure to overall IPV and 4 World Health Organization–designated IPV domains: physical, psychological and emotional, sexual, and controlling behaviors. Univariate meta-analyses using a random effects model were used to calculate the standardized mean differences (SMDs) and 95% CIs for each IPV outcome. The Grading of Recommendations Assessment, Development, and Evaluation method was used to rate the certainty of findings.

**Results:**

Overall, 10 randomized clinical trials met inclusion criteria, with a total of 16 136 participants, of whom 98% identified as women, with a mean age of 28.9 years. Compared with no intervention, participation in microfinance was associated with lower rates of psychological and emotional violence (SMD, 0.87; 95% CI, 0.80-0.95; *I*^2^ = 46%; high certainty), sexual violence (SMD, 0.76; 95% CI, 0.63-0.90; *I*^2^ = 44%; low certainty), and controlling behaviors (SMD, 0.82; 95% CI, 0.74-0.92; *I*^2^ = 54%; high certainty). There was no significant association with physical violence (SMD, 0.89; 95% CI, 0.76-1.04; very-low certainty).

**Conclusions and Relevance:**

This systematic review and meta-analysis of microfinance interventions found a reduction in exposure to psychological and emotional IPV as well as controlling behaviors among participants receiving microfinance interventions, with high certainty evidence. Further work is needed to evaluate which types of microfinance interventions are most effective at reducing the various forms of IPV.

## Key Points

**Question:**

Are microfinance interventions associated with reduced exposure to intimate partner violence?

**Findings:**

This systematic review and meta-analysis including 10 randomized clinical trials with 16 136 participants identified statistically significant reductions in psychological and emotional intimate partner violence, controlling behaviors, and sexual violence associated with microfinance interventions.

**Meaning:**

This systematic review and meta-analysis found that microfinance interventions were associated with reductions in some forms of intimate partner violence.

## Teaser

This systematic review and meta-analysis evaluates the associations between microfinance interventions and exposure to intimate partner violence.

## Introduction

The World Health Organization (WHO) defines intimate partner violence (IPV) as any behavior causing physical, psychological, or sexual harm to individuals within an intimate relationship.^1^ Women experience high exposure to IPV, with 27% of ever-partnered women between the ages of 15 to 49 years having experienced IPV in their lifetimes, with notably higher prevalence within certain low- and middle-income countries.^2^ Such violence is associated with profound short- and long-term physical and psychological consequences,^3,4,5^ from chronic pain disorders to risks of sexually transmitted infections, including HIV.^3,5^ IPV can also lead to various forms of mental illness^6,7,8,9,10,11,12,13^ as well as increased risk of mortality; more than 38% of all homicides of women are estimated to be committed by an intimate partner.^14^ Beyond the numerous direct health consequences, the societal and economic costs of IPV are substantial.^15^

A broad range of interventions have been implemented to reduce the risk of further IPV victimization, with varied success.^16^ Across the various forms of IPV, evidence supports interventions that empower women, increase social capital, reduce community and cultural acceptability of IPV, improve the quality of relationships, promote equitable gender norms, and improve economic well-being.^17^ Economic empowerment, in particular, may be an especially powerful intervention in resource limited settings where poverty may be a more prominent driver of IPV.^2,18^

The association between financial status and IPV is multipronged and nuanced. Power dynamics within a relationship often parallel financial power, which, because of masculine-centric gender norms in many contexts, often results in the disempowerment of women, compounding other gender norms (eFigure 1 in Supplement 1).^19,20^ Microfinance interventions were developed as an antipoverty strategy in low-income countries for individuals without access to traditional financial services (eg, loans, lines of credit, and savings accounts). Prior systematic reviews have explored the associations of microfinance interventions with various health metrics, with some findings suggesting potential health benefits.^21,22,23^ Microfinance interventions may be uniquely suited to addressing IPV in low- and middle-income settings by simultaneously alleviating financial, social, and cultural drivers of IPV in low-income settings by empowering survivors of violence directly.^24^

The IMAGE trial in South Africa combined microfinance interventions with HIV and gender-norm training and demonstrated notable success: participants’ past-year experiences of physical and sexual violence was reduced by 55%.^25,26^ Since then, several randomized clinical trials (RCTs) of microfinance interventions have been performed in other settings, with varying results. Some evidence suggests that the effect of microfinance may differ by type of violence (eg, physical, sexual, emotional).^25^ In addition, some concern has been raised that microfinance programs may actually increase risk of future IPV.^27^ To better understand those discrepant findings, we conducted a systematic review and meta-analysis of RCTs evaluating whether microfinance interventions reduce exposure to various forms of IPV.

## Methods

### Literature Search, Study Selection, and Data Extraction

This systematic review and meta-analysis was conducted in accordance with the Preferred Reporting Items for Systematic Reviews and Meta-analyses (PRISMA) reporting guideline.^28^ The protocol was registered in the International Prospective Register of Systematic Reviews (PROSPERO identifier: CRD42022353065).

To evaluate the effectiveness of microfinance interventions on reducing exposure to IPV in global settings, we developed a search strategy^29^ in consultation with an expert librarian. On August 3, 2022, we searched the following online databases using the database inception as the starting point to capture all articles within that database: PubMed, CINAHL, Embase, Web of Science, EconLit, African Index Medicus (AIM), Index Medicus for the Eastern Mediterranean Region (IMEMR), Index Medicus for the South-East Asia Region (IMSEAR), Latin America and the Caribbean Literature on Health Sciences (LILACS), and Western Pacific Index Medicus (WPRO). Full search strategies are presented in the eAppendix in Supplement 1. We then searched the reference lists of included articles to identify additional eligible studies. We included RCTs and cluster-randomized trials that enrolled individuals undergoing a microfinance intervention compared with a control (defined as no intervention, care as usual, or other services without microfinance intervention) and evaluated the primary outcome of experience of IPV. We did not search databases of abstracts, working papers, governmental documents, or program evaluations.

The outcome of IPV was defined according to the WHO definition as any form of physical, psychological, or sexual abuse or controlling behaviors by an intimate partner or former partner.^30^ Given its various forms, we evaluated 5 outcomes: overall IPV, physical abuse, psychological and emotional abuse, sexual abuse, and controlling behaviors.^2^ Studies using composite scales (eg, female empowerment index) without direct measurement of one of the predefined outcomes were excluded. Microfinance interventions were defined as microcredit (including cash and asset transfers) or microloan programs aimed at reducing poverty (eTable in Supplement 1). No language restrictions were used during title and abstract searching; however, we excluded full-text articles not available in English.

The articles generated by our search were aggregated using Covidence software. All authors independently screened identified titles and abstracts for study inclusion eligibility. Full texts were then independently reviewed by all authors for study eligibility. Discrepancies among reviewers was adjudicated by consensus.

All authors independently extracted the following data from the included articles using a prespecified abstraction form: study country or countries, study design, sample size, population characteristics (age and gender distribution), measurement tool used, qualitative description of the intervention, conditions of the control group, and effect estimates with 95% CIs, SEs, or SDs (eTable in Supplement 1). Effect estimates from the most adjusted model were used.

### Risk of Bias Assessment

To assess the risk of bias within the included articles, 2 reviewers (R.O. and Q.T.) independently evaluated each study using the appropriate Cochrane Risk of Bias Tools for RCTs.^31,32^ The following domains of each of the primary studies were assessed: random sequence generation, allocation concealment, blinding of study participants, incomplete outcome data, selective reporting, and other biases (eFigure 3 in Supplement 1). We assessed the certainty of evidence using the Grading of Recommendations Assessment, Development, and Evaluation (GRADE) method (ie, high, moderate, low, and very-low certainty).^33^

### Statistical Analysis

We report summary statistics of the included studies. When mean age was not available, we estimated mean age from provided median, range, and sample size.^34^ Using the extracted estimates of effect, we calculated the Cohen *d* (reported as standardized mean difference [SMD]) to standardize the estimates accounting for the use of different types of survey instruments and measurements (eTable in Supplement 1). We conducted a series of univariate meta-analyses using a random-effects model to calculate the pooled estimate and 95% CI for each outcome. We assessed for between-study heterogeneity by evaluating the *Q* statistic and *I*^2^ metrics of the model. We assessed for publication bias via funnel plot analysis and by Egger tests. We investigated heterogeneity by performing subgroup analyses for each domain of IPV by type of microfinance intervention and Cochrane risk of bias assessment. Further, we performed a leave-1-out sensitivity analysis, or sequential meta-analyses, using a random-effects model excluding 1 study at a time for all permutations.

All tests were 2-sided, and statistical significance was based on the 95% CIs excluding the null. The calculation of the Cohen *d* was performed using Comprehensive Meta-Analysis software version 3 (Biostat). All other analyses were performed using Stata software version 17.0 (StataCorp). Data were analyzed from August 6 to September 10, 2022.

## Results

**Figure 1.**
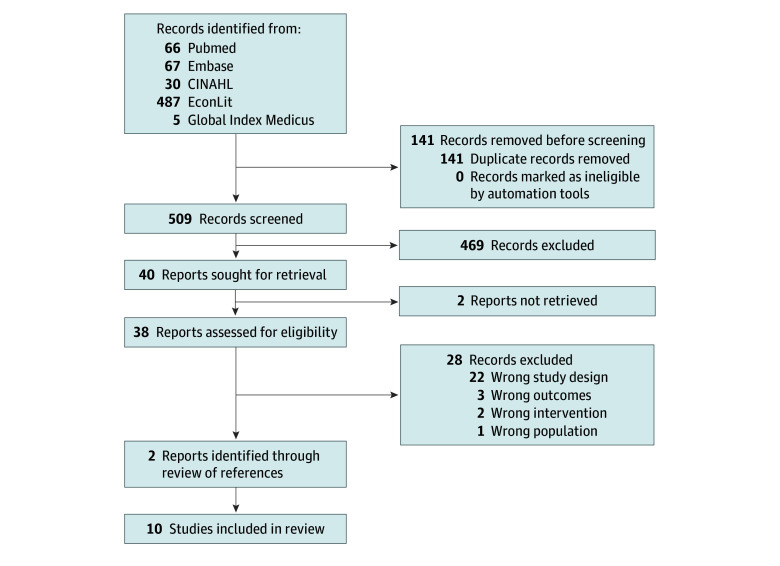
Flowchart for Study Identification

Our search identified 509 records, of which 40 were sought for retrieval (Figure 1). Of those, 8 studies met our inclusion criteria, while an additional 2 studies were identified from references of included studies. The 10 studies included^25,35,36,37,38,39,40,41,42,43^ were from 9 low-income countries according to the World Bank-classification (eFigure 2 in Supplement 1). Seven studies were cluster-randomized trials,^25,35,36,37,41,42,43^ and 3 studies^38,39,40^ were individually randomized clinical trials. From the 10 studies included, there were 16 136 total participants, of whom 98% identified as women. The pooled mean age of the study populations was 28.9 years.

### Study Characteristics

Nine studies^25,35,36,37,38,39,40,42,43^ (90%) included only women, and 8 studies^25,35,36,37,38,40,42,43^ (80%) included only women who were partnered or married, as these were considered the population at risk for IPV (eTable in Supplement 1). Three studies^35,36,37^ included mothers only, and all 3 were based on government-funded cash transfer programs. While most studies included predominantly young and middle-aged women, 1 study^39^ focused on adolescent girls and young women enrolled in high school programs.

Microfinance interventions included group microloan and microsavings models, provision of microcredits (including livestock), as well as conditional and unconditional cash transfers (Table 1). Six studies included a training component as part of the microfinance intervention: 4 studies^40,42,43,44^ included financial and business skills training, 1 study^35^ included health and nutrition education, and 1 study^25^ included gender and HIV training. Five studies provided unconditional^35,36,37,43^ or conditional^39^ cash transfers. Control groups for all studies provided no intervention, except Tankyard et al^38^ and Peterman et al,^37^ which provided limited health services (3 optional clinic visits and HIV and herpes simplex virus-2 testing, respectively) to the intervention and control groups.

**Table 1.** Characteristics of Included Studies Evaluating the Association of Microfinance With IPV

Source	Country	Study design	No.			Mean age, y	Intervention	Sessions^b^	Length, mo	Comparator	Outcomes	Timing of measurement of study outcomes
			Clusters	Participants in treatment/control^a^	Women/men^a^							
Pronyk et al,^25^ 2006	South Africa	Cluster RCT	8	430/430	860/0	41	Group loan program and gender/HIV training	10	12-15	No intervention	Any IPV (physical or sexual), controlling behaviors	12 mo
Hidrobo et al,^36^ 2013	Ecuador	Cluster RCT	118	1564/790	2354/0	23.6	Unconditional cash transfer	NA	36	No intervention	Physical IPV, psychological IPV, controlling behaviors	Any prior or current
Green et al,^43^ 2015	Uganda	Cluster RCT	120	896/904	1488/246	27.3	Unconditional cash transfer and business training	9	6	No intervention	Any IPV, controlling behaviors	8 mo
Glass et al,^41^ 2017	Democratic Republic of Congo	Cluster RCT	10	330/589	701/131	39.5	Microcredit (piglet, palm kernel) and business training	36	18	No intervention	Physical IPV, psychological IPV, and sexual IPV	3 mo
Peterman et al,^37^ 2017	Zambia	Cluster RCT	90	796/821	1617/0	28.2	Unconditional cash transfer, HIV and HSV-2 testing	NA	36	HIV and HSV-2 testing	Physical IPV	12 mo
Kilburn et al,^39^ 2018	South Africa	RCT	NA	1225/1223	2448/0	15.3	Conditional cash transfer (on ≥80% school attendance)	NA	36	No intervention	Physical IPV	12 mo
Tankyard et al,^38^ 2019	Colombia	RCT	NA	1364/436	1800/0	33.3	Microsavings, microcredit, and 3 free, optional health visits	NA	18	3 free, optional health visits	Any IPV, physical IPV	12 mos
Gibbs et al,^40^ 2020	Afghanistan	RCT	NA	747/714	1461/0	24.5	Microfinance and business training	88	12	No intervention	Physical IPV, psychological IPV	12 mo
Heath et al,^35^ 2020	Mali	Cluster RCT	39	1188/1550	1550/0	32	Unconditional cash transfer and health, nutrition training	12	23-27	No intervention	Physical IPV, psychological IPV, controlling behaviors	12 mo
Jalal et al,^42^ 2021	Bangladesh	Cluster RCT	261	767/560	1327/0	24.4	Microfinance and business training and support	NR	18	No intervention	Any IPV, physical IPV, psychological IPV, sexual IPV, controlling behaviors	12 mo

Abbreviations: HSV, herpes simplex virus; IPV, intimate partner violence; NA, not applicable; NR, not reported; RCT, randomized clinical trial.

^a^ Sample sizes for treatment and control groups are provided in accordance with study protocol, eg, intention-to-treat vs per-protocol analysis; due to attrition, gender and age distribution may not include full original sample. Age and gender distributions provided for relevant groups and subgroups (eg, women) analyzed for study outcome of interest.

^b^ When training component included in intervention, the approximate number of sessions offered per participant.

Eight studies^35,36,38,39,40,41,42,43^ (80%) assessed IPV outcomes using the standard or an adapted version of the WHO Violence Against Women Survey Instrument (eTable in Supplement 1).^44^ Of the various outcomes, 4 studies^25,38,42,43^ reported on any IPV, 7 studies^35,37,38,39,40,41,42^ reported on physical IPV, 5 studies^35,36,40,41,42^ reported on psychological IPV, 2 studies^41,42^ reported on sexual IPV, and 5 studies^25,35,36,42,43^ reported on controlling behaviors. Despite the lack of ability to blind participants from interventions, 6 trials^25,36,35,38,39,43^ (60%) were found to have a low risk of bias (eFigure 4 in Supplement 1). Two studies^40,41^ (20%) were found to have some concerns, and 2 studies^37,42^ (20%) had high risk of bias. The main areas of concern were regarding inconsistencies in measurement of outcomes and missing outcome data.

### Overall IPV

Four trials^25,38,42,43^ including 5787 individuals evaluated the impact of microfinance interventions on overall IPV. The pooled SMD was 0.87 (95% CI, 0.69-1.09; low-certainty evidence), indicating a potential for moderate benefit or slight harm. There was serious inconsistency (*I*^2^ = 83.7%) in the study outcome of overall IPV, which was not explained with the planned subgroup analyses by intervention type nor risk of bias assessment (Table 2; eFigure 4 in Supplement 1).

**Table 2.** Summary of Findings for the Certainty of Our Primary Outcomes Using the GRADE Method

Outcome	Certainty assessment							Participants, No.		Certainty	
	Studies, No.	Study design	Risk of bias	Inconsistency	Indirectness	Imprecision	Other considerations	Microfinance interventions	Control	Absolute (95% CI)	Ranking
Overall IPV	4	Randomized trials	Not serious	Serious	Serious	Serious	None	3453	2317	SMD 0.87 SD lower (0.69 lower to 1.09 higher)	Very low^a^
Physical abuse	7	Randomized trials	Not serious	Serious	Serious	Serious	None	3896	3792	SMD 0.88 SD lower (0.74 lower to 1.04 higher)	Very low^b^
Psychological and emotional abuse	5	Randomized trials	Not serious	Not serious	Serious	Not serious	None	2918	2547	SMD 0.87 SD lower (0.8 lower to 0.95 higher)	High^c^
Sexual abuse	2	Randomized trials	Not serious	Not serious	Not serious	Serious	None	976	934	SMD 0.76 SD lower (0.63 lower to 0.9 higher)	Low^d^
Controlling behaviors	5	Randomized trials	Not serious	Not serious	Serious	Not serious	None	3700	3070	SMD 0.82 SD lower (0.72 higher to 0.92 higher)	High^e^

Abbreviations: IPV, intimate partner violence; SMD, standardized mean difference.

^a^ Important inconsistency (*I*^2^ = 83.7%; *P* < .001) not explained by planned sensitivity analysis of microfinance intervention subtype (eFigure 4 in Supplement 1). Evidence was imprecise, as point estimates and CIs varied considerably.

^b^ Important inconsistency (*I*^2^ = 86.3%, *P* < .001), only partially explained by planned sensitivity analysis of microfinance intervention subtype (heterogeneity resolves for cash transfer interventions and co-delivery with health education; however heterogeneity increases for interventions delivered with business training, (*I*^2^ = 93%), eFigure 4 in Supplement 1). Some studies provided variable cointerventions (eg, nutrition training, school incentives, optional health visits) that limit the overall intervention directness, and could lead to a difference in outcome. The 95% CI includes no effect, and point estimates and CIs varied considerably.

^c^ One study^35^ provided a cointervention (eg, nutrition training); however, the leave-1-out sensitivity analysis did not alter the effect estimate when this study was removed.

^d^ Two represented countries (Bangladesh, Democratic Republic of Congo) with contexts that may not be directly comparable to other low-income countries. Pooled population size does not meet the optimal information size requirement, leading to concern for imprecision.

^e^ Some important indirectness as cointerventions varied (eg, cash transfers, health training). Planned sensitivity analysis demonstrated significant group differences by intervention subtypes; addition of business training was associated with a significant reduction in psychological and emotional IPV as well as controlling behaviors, while cash transfers alone were not associated with a significant reduction in psychological and emotional IPV.

### Physical IPV

Seven trials^35,37,38,39,40,41,42^ including 9505 individuals evaluated the impact of microfinance on physical IPV outcomes. Compared with no intervention, participation in a microfinance intervention was associated with an estimated decrease in physical IPV by 0.11 points (SMD, 0.89; 95% CI, 0.76-1.04; very-low certainty). There was notable inconsistency (*I*^2^ = 83.6%) and imprecision of intervention effects. Subgroup analysis by intervention type demonstrated that heterogeneity significantly reduced for cash transfer interventions (*I*^2^ = 0%) but increased for interventions combined with business training (*I*^2^ = 93.0%) (Table 2). Subgroup analysis by risk of bias (eFigure 4 in Supplement 1) found decreased heterogeneity (*I*^2^ = 0%) among the 2 studies^38,39^ deemed to have low risk of bias.

### Psychological and Emotional IPV

**Figure 2.**
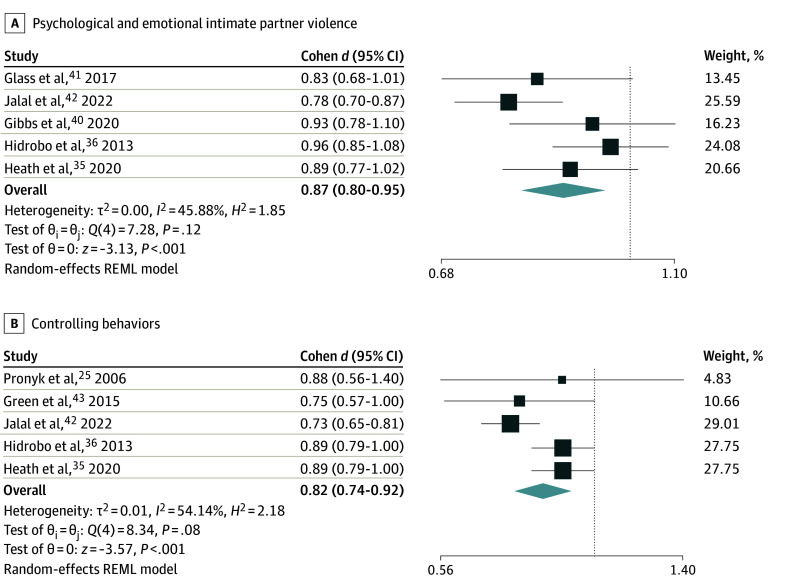
Meta-analysis of the Association of Microfinance Interventions With Different Domains of Intimate Partner Violence Boxes indicate *d*; whiskers, 95% CIs; diamonds, pooled effect sizes; REML, restricted maximum likelihood.

**Figure 3.**
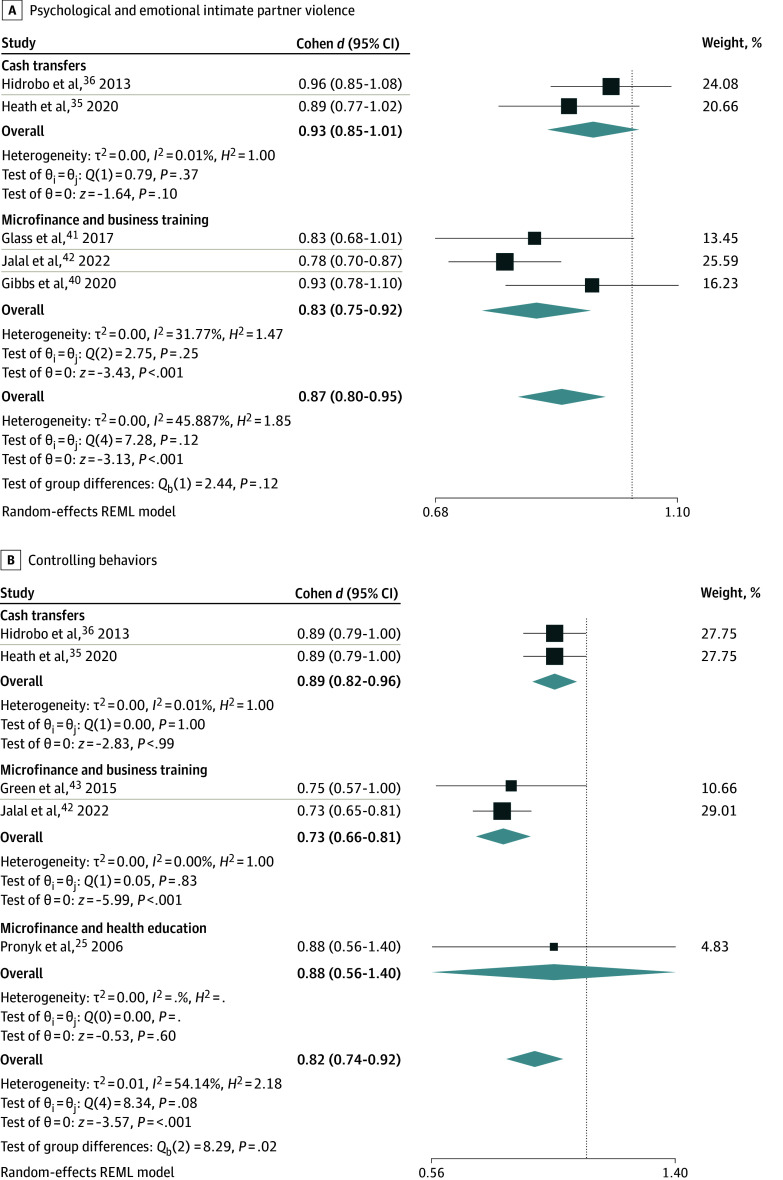
Subgroup Analyses by Type of Microfinance in Association With Different Domains of Intimate Partner Violence Boxes indicate *d*; whiskers, 95% CIs; diamonds, pooled effect sizes; REML, restricted maximum likelihood.

Five trials^35,36,40,41,42^ including 7611 individuals evaluated the impact of microfinance interventions on psychological/emotional IPV. Participation in a microfinance intervention was associated with reduction in psychological and emotional IPV by 0.13 points (SMD, 0.87; 95% CI, 0.80-0.95), with high certainty evidence (Figure 2A). Results were consistent across studies, estimates were precise, and evidence was direct. There was moderate heterogeneity (*I*^2^ = 45.9%), which decreased in the subgroup analysis by intervention type (Figure 3) for both cash transfers (*I*^2^ = 0%) and microfinance combined with business training (*I*^2^ = 32%). Psychological and emotional IPV was reduced further (SMD, 0.81; 95% CI, 0.71-0.92) among the group that received microfinance interventions with business training, compared with those who received cash transfers alone (SMD, 0.93; 95% CI, 0.84-1.01); however, the test of group differences was not significant (*P* = .12) (Figure 3). The subgroup analysis by risk of bias did not significantly change the results.

### Controlling Behaviors

Five trials^25,35,36,42,43^ of 7891 individuals evaluated the impact of microfinance programs on controlling behaviors. Microfinance interventions were associated with a significant decrease in controlling behaviors (SMD, 0.82; 95% CI, 0.74-0.92), with high certainty evidence (Figure 2B). There was moderate heterogeneity within the model (*I*^2^ = 54.1%). In the subgroup analysis, the association with controlling behaviors became more favorable, reducing to an SMD of 0.69 (95% CI, 0.59-0.79) among the group receiving microfinance interventions as well as business training, with heterogeneity less than 1% in both groups (Figure 3; eFigure 4 in Supplement 1). Among those receiving cash transfers alone, the effect size became less favorable (SMD, 0.88; 95% CI, 0.80-0.96). The difference in effect sizes was statistically significant (*P* for group difference = .02). Subgroup analyses by risk of bias did not significantly alter the results (eFigure 4 in Supplement 1).

### Sexual IPV

Compared with no intervention, microfinance interventions were associated with reduced exposure to sexual forms of IPV (SMD, 0.76; 95% CI, 0.63-0.90) with low-certainty evidence. Evidence was deemed low precision, as the sample size did not meet the optimal information size to reliably assess sexual violence as an outcome.

### Sensitivity and Publication Bias Analyses

Our sensitivity analyses revealed no significant changes in the significance of the pooled estimates for any of the domains of IPV (eFigure 4 in Supplement 1). Notably, for sexual forms of IPV, we could not perform a sensitivity analysis because there were only 2 eligible studies. Our analyses for publication bias revealed no evidence of publication bias for any domain of IPV. We could not calculate Egger test for sexual IPV because there were only 2 studies. Funnel plots for all IPV domains are reported in eFigure 5 in Supplement 1.

## Discussion

This systematic review and meta-analyses of 10 RCTs of 16 136 individuals evaluated the associations of various microfinance interventions with several domains of IPV globally. Our results highlight 2 salient findings. First, microfinance interventions were associated with a reduction in 2 forms of IPV: psychological and emotional violence and controlling behaviors. Second, despite those findings, there remains a paucity of reports studying the impacts of various types of microfinance interventions on different forms of IPV.

Poverty is a key driver associated with IPV,^45,46,47^ and is associated with several indirect drivers of IPV, including worse educational outcomes and higher exposure to childhood abuse and neglect.^45^ Microfinance interventions may be uniquely positioned to address those drivers. The included studies evaluated several models of microfinance programs, including direct cash and asset transfers, microloans, and microsavings; several included training sessions with variable session lengths and foci. Despite that variability, the fundamental output of those interventions was improved economic and social standing, which was likely a key component of the observed associated reduction in IPV. Furthermore, observed reductions were persistent and significant (for psychological and emotional IPV and controlling behaviors) despite heterogeneity in microfinance interventions.

Notably, drivers of IPV beyond poverty include patriarchal gender norms, conflict and postconflict settings, poor mental health, and substance use disorders.^45,46,47,48,49^ Poverty alleviation may have pleotropic effects on a number of drivers, but it is unlikely to alleviate all drivers of IPV. Furthermore, the drivers and thus effective mitigators of violence may vary based on the type of IPV, which in turn may vary with the socioeconomic status of the population. For example, armed conflict may be a stronger driver associated with sexual violence compared with other forms of violence.^50^ Notably, our assessment of sexual IPV was underpowered to detect an association, given that only 2 studies evaluated that domain independently. However, what is further evident from our findings is that economic empowerment in the form of microfinance interventions was associated with reductions in IPV across multiple domains, suggesting a potentially dominant role of poverty and status in IPV. Such an outcome may be attributable to increased bargaining power within abusive relationships, financial independence enabling survivors to leave violent living situations, and reduced financial stress.^51^

However, microfinance interventions were not associated with significant reductions in physical IPV. Prior research has suggested that women with higher financial earnings than their partners may experience increased physical IPV,^52^ which was not observed in our study. Thus, the association of microfinance interventions with physical IPV may be nuanced, with some resultant protective associations (such as increased self-esteem and self-efficacy) and some risk factors (increased financial means). Supporting that hypothesis is the observation that the combined association of microfinance interventions with physical IPV had high heterogeneity. Future research should explore drivers specific to physical IPV. Interventions aiming to address physical IPV may require additional or supplemental approaches beyond microfinance programs.

Our subgroup analyses supported the combination of financial educational sessions with microfinance interventions in association with reducing psychological and emotional IPV and controlling behaviors. That finding is perhaps related to improved financial status among the participants who received financial education sessions compared with those who did not, thus conferring additional protective associations in the form of greater self-esteem and self-efficacy. In particular, the microfinance subgroup that received business training had the strongest association with a reduction in controlling behaviors. That may reflect transposition of the business skills learned to other settings. Future microfinance interventions should consider pairing interventions with financial education to augment the impact of their interventions. Additional consideration should also be given to pairing microfinance interventions with trainings that focus on gender equity and violence prevention, which was done by Pronyk et al.^25^ Such trainings might also consider including, where appropriate, the partners of the participants. However, our results were underpowered to assess the outcomes associated with the trainings to such detail. There are many possible future directions for expansion of paired training sessions, and more work is needed to assess which training sessions have the greatest effect on reducing IPV and other related health outcomes.

### Limitations

This study has some limitations. First, our study was limited in the number of studies included in the final analysis; however, the large sample sizes were above the optimal information size threshold^53^ for all outcomes of interest except sexual abuse, suggesting that outcomes were powered to detect the associations of the study intervention. Within the studies identified, 40% had moderate or high risk of bias, which was accounted for in the GRADE certainty of evidence assessment. Additionally, the socioeconomic contexts that drive IPV are likely nuanced and interrelated to the geographic locations in which they occur. Our study included trials predominantly from low- and middle-income countries, thus the generalizability of our findings may be imperfect; furthermore, the geographic regions in which the studies occurred may not be representative of all areas, specifically Southeast Asia where microfinance interventions have been substantially implemented. The strengths of our study lie in the large sample sizes, randomized clinical trial designs, and rigorous methodological approach (eg, GRADE) used to evaluate the potential effects of microfinance interventions on study outcomes, supporting the accuracy and reliability of our results. Given the global need to address IPV in all of its forms, we feel the limitations of our study do not negate the importance of our findings.

## Conclusions

In this systematic review and meta-analysis of 10 randomized clinical trials from 9 different countries, we found that microfinance interventions were associated with reductions in psychological and emotional IPV as well as controlling behaviors. Further work should evaluate the impact of microfinance interventions on the risk for sexual IPV. Overall, much more work is needed to combat IPV in all of its forms, and our results suggest that microfinance may be a useful tool to do so, warranting further expansion in other settings.

## Notes

Supplement 1. **eAppendix.** Search Strategy **eFigure 1. **Evidence Framework for How Microfinance Interventions Might Impact the Drivers of Intimate Partner Violence **eTable. **Study Intervention Details and Outcome Measurement Information **eFigure 2. **Global Distribution of Studies Included in Meta-analysis **eFigure 3. **Risk of Bias Assessment of the 10 Studies Included in Meta-analysis **eFigure 4. **Results of the Sensitivity Analyses for Each Domain of Intimate Partner Violence **eFigure 5. **Galbraith Plots and Funnel Plots Assessing for Heterogeneity and Publication Bias

Supplement 2.
Data Sharing Statement
