# Supplementary material for: Assessment of Microfinance Interventions and Intimate Partner Violence: A Systematic Review and Meta-analysis
Source: JAMA Netw Open. 2023 Jan 27;6(1):e2253552. doi: 10.1001/jamanetworkopen.2022.53552 (PMC12543409; doi:10.1001/jamanetworkopen.2022.53552)
Supplement: Supplement 1. — eAppendix. Search Strategy eFigure 1. Evidence Framework for How Microfinance Interventions Might Impact the Drivers of Intimate Partner Violence eTable. Study Intervention Details and Outcome Measurement Information eFigure 2. Global Distribution of Studies Included in Meta-analysis eFigure 3. Risk of Bias Assessment of the 10 Studies Included in Meta-analysis eFigure 4. Results of the Sensitivity Analyses for Each Domain of Intimate Partner Violence eFigure 5. Galbraith Plots and Funnel Plots Assessing for Heterogeneity and Publication Bias [file jamanetwopen-e2253552-s001.pdf]

Supplemental Online Content

Allan-Blitz LT, Olson R, Tran Q. Assessment of microfinance interventions and intimate partner violence: a systematic review and meta-analysis. *JAMA Netw Open*. 2023;6(1):e2253552. doi:10.1001/jamanetworkopen.2022.53552

eAppendix. Search Strategy
eFigure 1. Evidence Framework for How Microfinance Interventions Might Impact the Drivers of Intimate Partner Violence
eTable. Study Intervention Details and Outcome Measurement Information
eFigure 2. Global Distribution of Studies Included in Meta-analysis
eFigure 3. Risk of Bias Assessment of the 10 Studies Included in Meta-analysis
eFigure 4. Results of the Sensitivity Analyses for Each Domain of Intimate Partner Violence
eFigure 5. Galbraith Plots and Funnel Plots Assessing for Heterogeneity and Publication Bias

This supplemental material has been provided by the authors to give readers additional information about their work.

© 2023 Allan-Blitz LT et al. *JAMA Network Open.*

eAppendix. Search Strategy

Database: **PubMed**
Interface: **National Library of Medicine**
Date of search: **3 August 2022**
Developed by: **Lao Allan-Blitz, Paul Bain, Rose Olson, Quang Tran**
Restrictions**: None**
Results: **66**

("Spouse Abuse"[Mesh] OR "Intimate Partner Violence"[Mesh] OR "Domestic Violence"[Mesh] OR "Battered Women"[Mesh] OR domestic violence[tiab] OR domestic abuse[tiab] OR partner violence[tiab] OR partner abuse[tiab] OR gender based violence[tiab] OR sexual abuse[tiab] OR sexual assault*[tiab] OR sexual violence[tiab] OR violence against women[tiab] OR wife-beat*[tiab] OR battered women[tiab] OR marital violence[tiab] OR spousal violence[tiab] OR beaten women[tiab])
AND
(microfinance*[tiab] OR micro-finance*[tiab] OR microcredit*[tiab] OR micro-credit*[tiab] OR microsaving*[tiab] OR micro-saving*[tiab] OR microloan*[tiab] OR micro-loan*[tiab])

Database: **Embase**
Interface: **National Library of Medicine**
Date of search: **3 August 2022**
Developed by: **Lao Allan-Blitz, Paul Bain, Rose Olson, Quang Tran**
Restrictions**: None**
Results**: 67**

1. 'partner violence'/exp OR 'domestic violence'/de OR 'battered woman'/exp OR ('domestic violence' OR 'domestic abuse' OR 'partner violence' OR 'partner abuse' OR 'gender based violence' OR 'sexual abuse' OR 'sexual assault*' OR 'sexual violence' OR 'violence against women' OR 'wife-beat*' OR 'battered women' OR 'marital violence' OR 'spousal violence' OR 'beaten women'):ab,ti,kw

2. ('microfinance*' OR 'micro-finance*' OR 'microcredit*' OR 'micro-credit*' OR microsaving* OR 'micro-saving*' OR microloan* OR 'micro-loan*'):ab,ti,kw

---

Database: **Web of Science Core Collection**
Interface: **Web Of Science**
Date of search: **3 August 2022**
Developed by: **Lao Allan-Blitz, Paul Bain, Rose Olson, Quang Tran**
Restrictions**: None**

© 2023 Allan-Blitz LT et al. *JAMA Network Open.*

Results**: 184**

TS=("domestic violence" OR "domestic abuse" OR "partner violence" OR "partner abuse" OR "gender based violence" OR "sexual abuse" OR "sexual assault*" OR "sexual violence" OR "violence against women" OR "wife-beat*" OR "battered women" OR "marital violence" OR "spousal violence" OR "beaten women")

TS=("microfinance*" OR "micro-finance*" OR "microcredit*" OR "micro-credit*" OR microsaving* OR "micro-saving*" OR microloan* OR "micro-loan*")

Database: **EconLit**
Interface: **ProQuest**
Date of search: **3 August 2022**
Developed by: **Lao Allan-Blitz, Paul Bain, Rose Olson, Quang Tran**
Restrictions**: Search anywhere except full texts**
Results**: 487**

("domestic violence" OR "domestic abuse" OR "partner violence" OR "partner abuse" OR "gender based violence" OR "sexual abuse" OR "sexual assault*" OR "sexual violence" OR "violence against women" OR "wife-beat*" OR "battered women" OR "marital violence" OR "spousal violence" OR "beaten women")

("microfinance*" OR "micro-finance*" OR "microcredit*" OR "micro-credit*" OR microsaving* OR "micro-saving*" OR microloan* OR "micro-loan*" OR “microlend*” OR “micro-lend*”)

Database:**CINAHL**
Interface: **EBSCOhost**
Date of search: **3 August 2022**
Developed by: **Lao Allan-Blitz, Paul Bain, Rose Olson, Quang Tran**
Restrictions**: None**
Results: **30**

MH domestic violence OR MH domestic abuse OR MH violence against women OR MH intimate partner violence OR MH partner violence OR MH partner abuse OR MH gender based violence OR MH gender based abuse OR MH domestic violence OR MH domestic abuse OR MH sexual assault OR MH sexual abuse OR TI domestic violence OR AB domestic violence OR TI domestic abuse OR AB domestic abuse OR TI partner violence OR AB partner violence OR TI partner abuse OR AB partner abuse OR TI gender based violence OR AB gender based violence OR TI sexual abuse OR AB sexual abuse OR TI sexual assault* OR AB sexual assault* OR TI sexual violence OR AB sexual violence OR TI violence against women OR AB violence against women OR TI wife-beat* OR AB wife-beat* OR TI battered women OR AB battered women OR TI marital

© 2023 Allan-Blitz LT et al. *JAMA Network Open.*

violence OR AB marital violence OR TI spousal violence OR AB spousal violence OR TI beaten women OR AB beaten women

TI microfinance* OR AB micro-finance* OR TI microcredit* OR AB micro-credit* OR TI microsaving* OR AB microsaving* OR TI micro-saving* OR AU micro-saving* OR TI microloan* OR AB microloan* OR TI micro-loan* OR AB micro-loan* OR TI microlend* OR AB microlend* OR TI micro-lend* OR AB micro-lend*

Databases: **African Index Medicus (AIM), Index Medicus for the Eastern Mediterranean Region (IMEMR), Index Medicus for the South-East Asia Region (IMSEAR), Latin America and the Caribbean Literature on Health Sciences (LILACS), and Western Pacific Index Medicus (WPRO)**
Interface: **Global Index Medicus**
Date of search: **3 August 2022**
Developed by: **Lao Allan-Blitz, Paul Bain, Rose Olson, Quang Tran**
Restrictions**: None**
Results**: 5**

("domestic violence" OR "domestic abuse" OR "partner violence" OR "partner abuse" OR "gender based violence" OR "sexual abuse" OR "sexual assault" OR "sexual violence" OR "violence against women" OR "wife-beat" OR "battered women" OR "marital violence" OR "spousal violence" OR "beaten women")

(microfinance* OR "micro-finance" OR microcredit* OR "micro-credit" OR microsaving* OR "micro-saving" OR microloan* OR "micro-loan")

© 2023 Allan-Blitz LT et al. *JAMA Network Open.*

eFigure 1. Evidence Framework for How Microfinance Interventions Might Impact the Drivers of Intimate Partner Violence

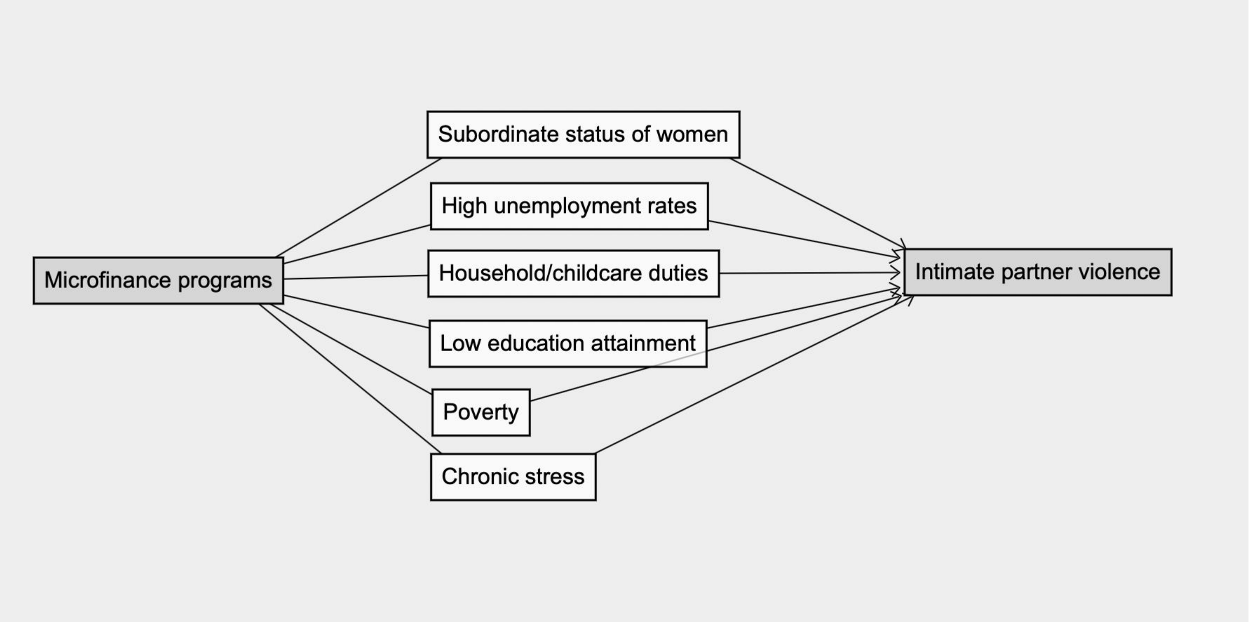

© 2023 Allan-Blitz LT et al. *JAMA Network Open.*

eTable. Study Intervention Details and Outcome Measurement Information

Study	Subgroup characteristics	In depth intervention description	Outcome measurement tool	Outcome variable type	Tool characteristics
Pronyk et al	Married women	The microfinance intervention was implemented by a large, experienced, established organization (Small Enterprise Foundation). The microfinance intervention included a group loan model, where women were encouraged to participate in individual businesses and groups of five women who guaranteed each others' loans. One loan center included approximately 40 women (8 groups of five) who met every two weeks.<br><br>Sisters for life (SFL) was a 12–15-month training program implemented in concert with a separate microfinance intervention by a separate team. SFL included ten 1-hour sessions, and included topics of IPV, gender roles and attitudes, and HIV, with a focus on enhancing communication and leadership skills. It also included a community mobilization component where key women were selected for another week of leadership training to mobilize around IPV and HIV issues in their community.	Internally developed and validated survey instruments	Binary	Any IPV: 4- item survey (2 physical, 2 sexual) where "yes" to any question was a positive outcome<br><br>Controlling behaviors: 4-item survey, coded as above
Hidrobo et al	Married mothers	National social assistance program called "Bono de Desarrollo Humano (BDH)" of 100,000 sucre (approximately **$15 USD) per month** to poor mothers, specifically households in bottom two poverty quintiles according to the Sistema de Selección de Beneficiarios (SELBEN) index. Initially planned to have child education and healthcare conditions, but implemented as unconditional. Transfers were administered through the government and distributed through a large network of private banks.	Internally developed survey, adapted from WHO VAW survey instrument	Binary	Physical IPV: 2-item survey where "yes" to any was a positive outcome<br><br>Psychological IPV: 4-item survey coded as above<br><br>Controlling behavior: 3-item survey, coded as above
Green et al	Partnered women	The WINGS program included four days of business skills training, approximately **$150 USD**, and follow-up support. The training included skills in business planning, budgeting, and record keeping. Cash was disbursed in two installments and staff visited the clients approximately every six weeks for six months to monitor spending and provide advice.	Internally developed survey, adapted from WHO VAW survey instrument	Binary	Any IPV: 5-item scale on frequency of IPV experienced (2 physical, 2 emotional, 1 sexual); where responses of "often", "sometimes", or "rarely" were coded as a binary positive outcome<br><br>Controlling behaviors: 6-question scale, coded as above
Glass et al	Partnered men or women	In partnership with local microfinance organization PAIDEK and JHSON, a micro-'credit' was provided in the form of piglet, aged 2-4 months, and 50 kg of palm kernel to participants in exchange for an agreement to	Internally developed survey, adapted from WHO VAW survey instrument	Binary	Physical IPV: 7-item survey, where "yes" to any was a positive outcome

© 2023 Allan-Blitz LT et al. *JAMA Network Open.*

		build a pigpen, compost pit, and repayment through giving 2 offspring piglets to other members of the microfinance group. Trained staff also provided biweekly business skills trainings, advice on nutrition and care of livestock, support for group meetings, and veterinarian visits.			Psychological IPV: 3-item survey; coded as above<br><br>Sexual IPV: 2-item survey; coded as above
Peterman et al	Married or cohabitating females with children under age of 5	National unconditional cash transfer of **approximately $24 USD every 2 weeks** to the primary female adult in households with a child under the age of 5.	Internally developed survey	Binary	Physical: 1-item survey, where "yes" was positive outcome
Kilburn et al	Unmarried, non-pregnant females enrolled in high school	Monthly cash transfer to female high school student and their households conditional on >80% high school attendance. All participants visited monthly by staff for interview, HIV and HSV-2 testing (if negative at the previous visit), and pre and post-test HIV counselling.	WHO VAW survey instrument, physical violence subset	Binary	Physical: 6-item survey, where "yes" to any was positive outcome
Tankyard et al	Partnered women	Intervention included a free, no-fee personal savings account at a local bank, as well as 10,000 peso (**approximately $5 USD**) initial deposit, and subsequent deposits to the account were matched at a rate of 1/3 up to a limit. Support and incentives were given to open up account and encourage usage (e.g. reminder text messages, directions, small lotteries). All participants were given a voucher for three free health checkups at a health clinic, providing access to certain services including a medical checkup, serology, and a family planning visit.	Adapted version of the WHO VAW survey instrument	Ordinal	11-item scale, where any IPV included questions on physical, emotional, financial, and sexual violence. Scale was 0-11 where 1 point was given for each "yes" to answer question on the scale.
Gibbs et al	Married women	Weekly trainings of 90-180 minutes provided to groups of 25 women on business and numeracy skills. In addition, participants receive a **monthly** cash stipend of **approximately $10 USD,** introduction to savings mechanisms, and referrals to health, legal and financial services, and connections to other women.	Adapted version of the WHO VAW survey instrument	Binary	Physical IPV: 5-item scale on frequency of IPV experienced, where responses were coded as never, once, few, or many. Responses of once or more frequently were coded as a binary positive outcome<br><br>Psychological IPV: 7-item scale, coded as above.
Heath et al	Married mothers of children under age 5	National cash transfer program, where participants were provided 10,000 FCFA per month (approximately $18 USD/month, or 9% of the household's monthly consumption) paid every quarter over a 3-year period to pregnant women and children under 5. Intervention participants also received two training sessions per month conducted by nongovernmental organizations, open to other household members in addition to the head. Sessions were organized into groups of themes, with each group covered for a period of 6 months. Training sessions related to nutrition, health, and other topics.	WHO VAW survey instrument	Binary	Physical IPV: 6-item scale on frequency of IPV experienced, where responses were coded as never, once, few, or many. Responses of once or more frequently were coded as a binary positive outcome<br><br>Psychological IPV: 4-item scale, coded as above<br><br>Controlling behaviors: 7- item

© 2023 Allan-Blitz LT et al. *JAMA Network Open.*

					scale, coded as above
Jalal et al	Women	Intervention was the implemented by BRAC, a non-governmental organization and international leader in microfinance programs. The program was known as Challenging the Frontiers of Poverty Reduction—Targeting the Ultra-Poor (CFPR-TUP) and included an asset transfer (e.g. cow, goat, poultry, or non-farm assets worth approximately 6000 Bangladeshi taka or 70 USD), weekly stipends, skills training for business development, income savings, health care education, and social. After the 18-month period, participants are expected to graduate to BRAC's conventional microcredit program.	WHO VAW survey instrument	Ordinal	18-item survey on frequency of IPV experienced, where responses were coded as never, once, few, or many. which included five categories of violence: physical violence, psychological violence, controlling behaviors, sexual violence

eFigure 2. Global Distribution of Studies Included in Meta-analysis

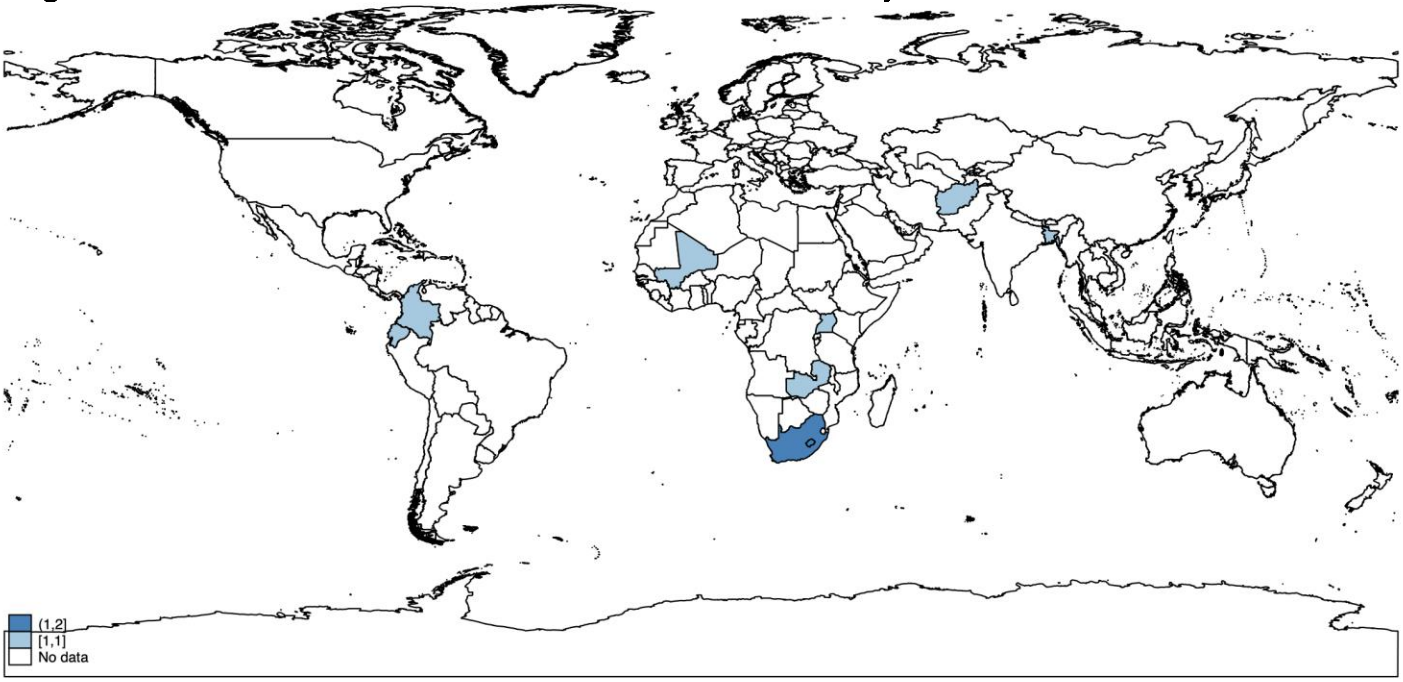

Figure 2 Legend: The map demonstrates the countries in which studies were conducted evaluating the impact of microfinance interventions on intimate partner violence. Darker shading indicate higher study density.

© 2023 Allan-Blitz LT et al. *JAMA Network Open.*

eFigure 3. Risk of Bias Assessment for the 10 Studies Included in the Systematic Review and Meta-analysis of the Impact of Microfinance Interventions on Intimate Partner Violence

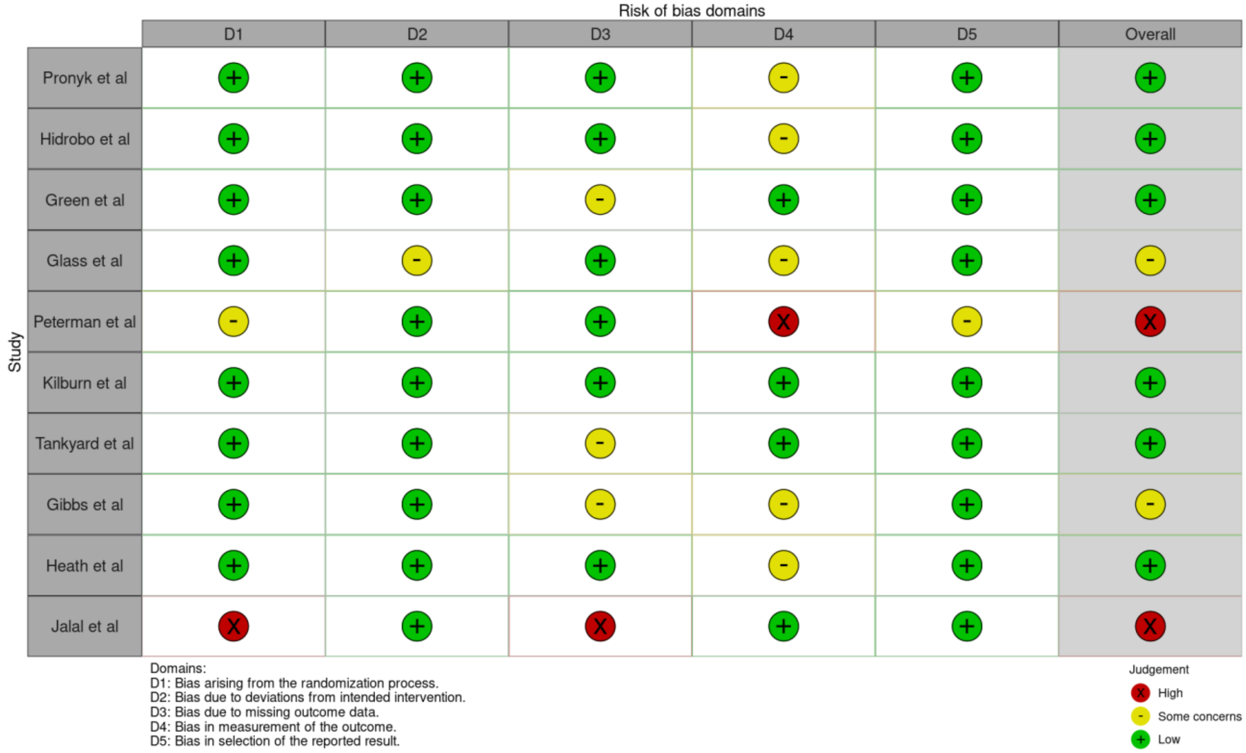

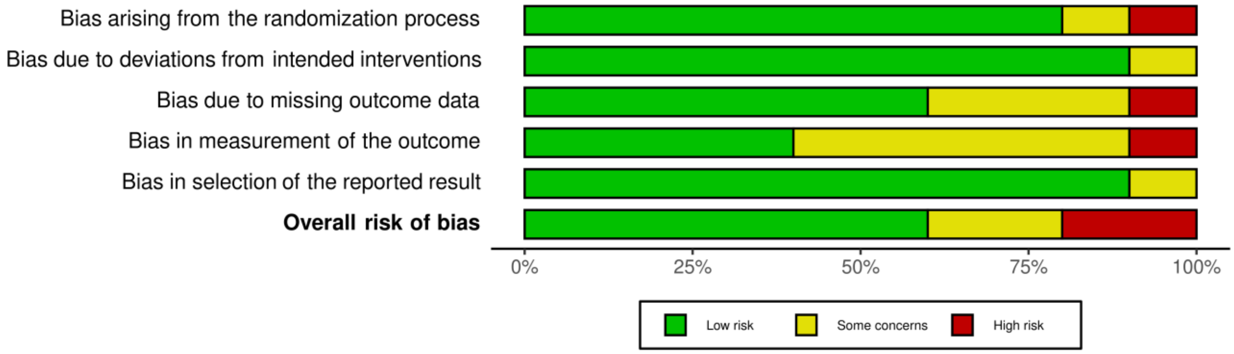

© 2023 Allan-Blitz LT et al. *JAMA Network Open.*

eFigure 4. Results of the Sensitivity Analyses for Each Domain of Intimate Partner Violence

eFigure 4a: Quality Index

Sub-Domain analysis by Cochrane's Risk of Bias Assessment for the impact on overall intimate partner violence

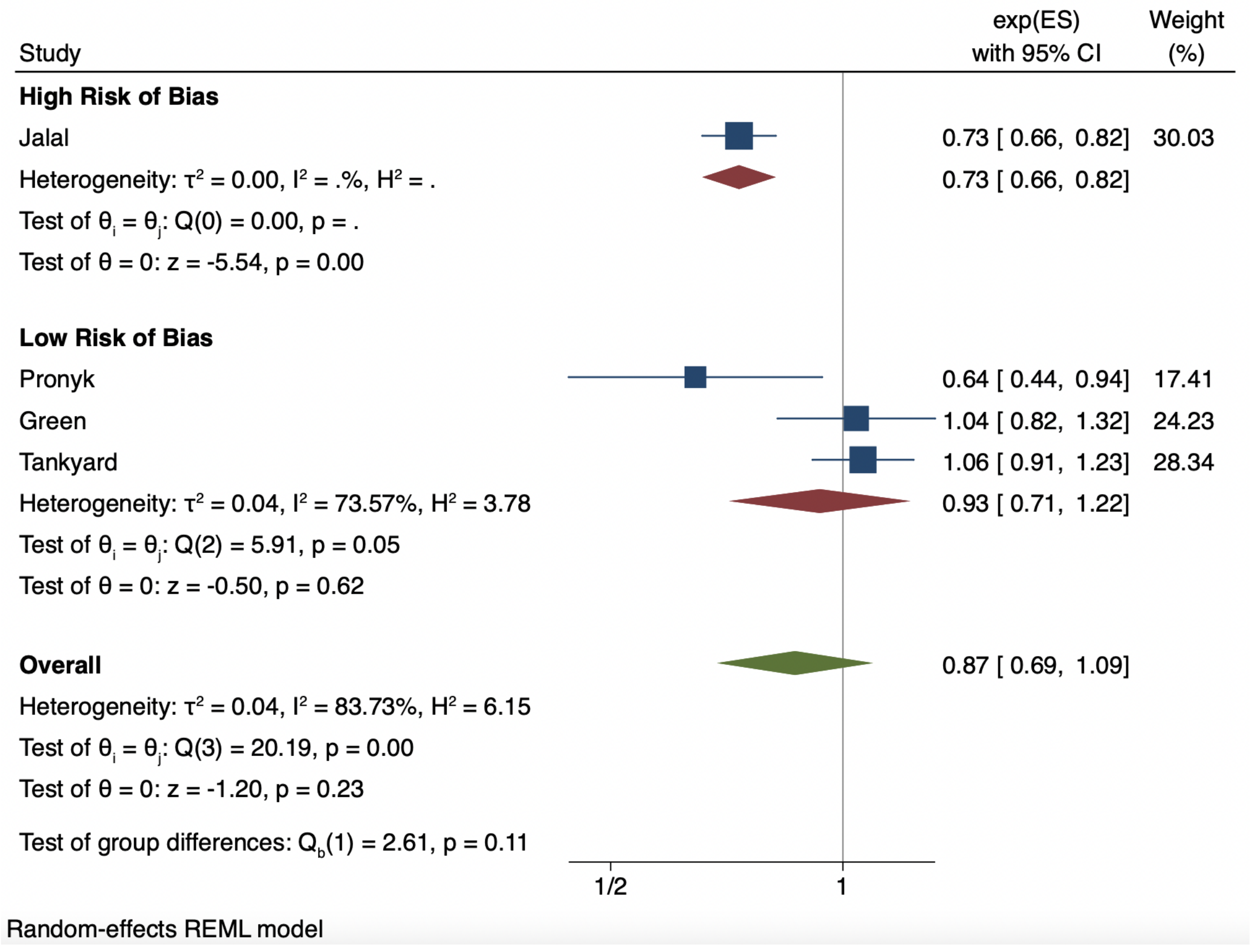

© 2023 Allan-Blitz LT et al. *JAMA Network Open.*

Sub-Domain analysis by Cochrane's Risk of Bias Assessment for the impact on physical intimate partner violence

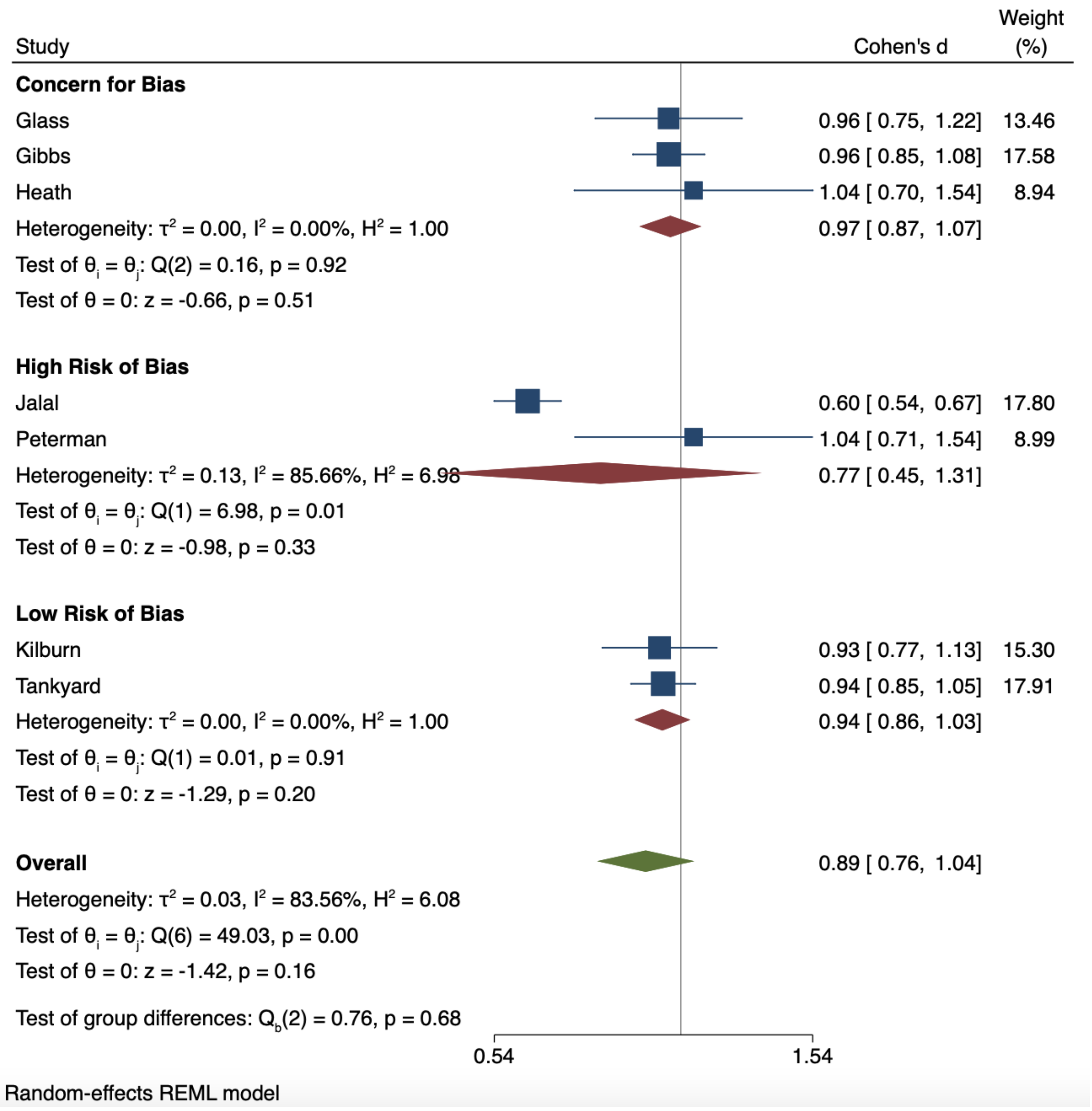

© 2023 Allan-Blitz LT et al. *JAMA Network Open.*

Sub-Domain analysis by Cochrane's Risk of Bias Assessment for the impact on psychological and emotional intimate partner violence

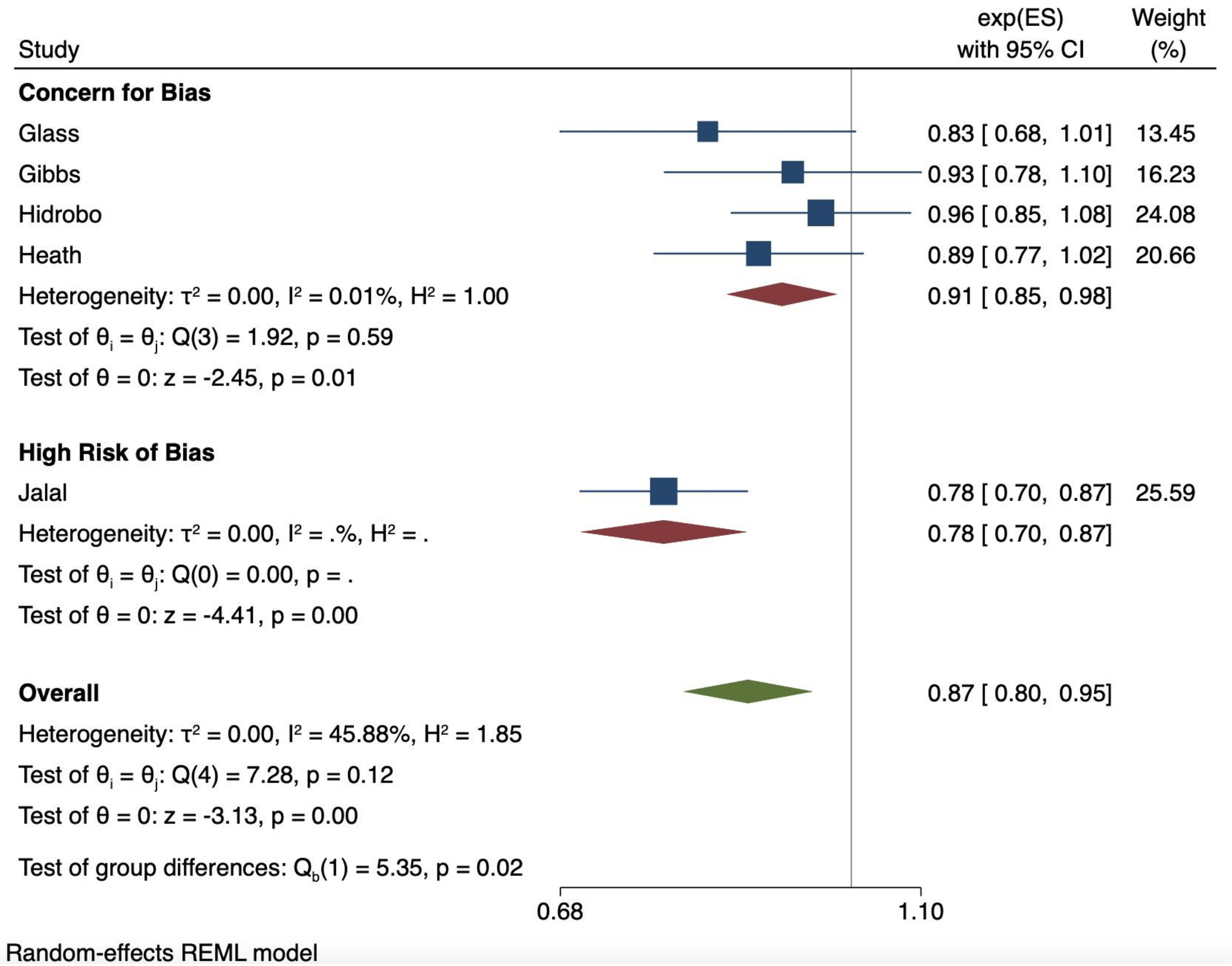

© 2023 Allan-Blitz LT et al. *JAMA Network Open.*

Sub-Domain analysis by Cochrane's Risk of Bias Assessment for the impact on controlling behaviors

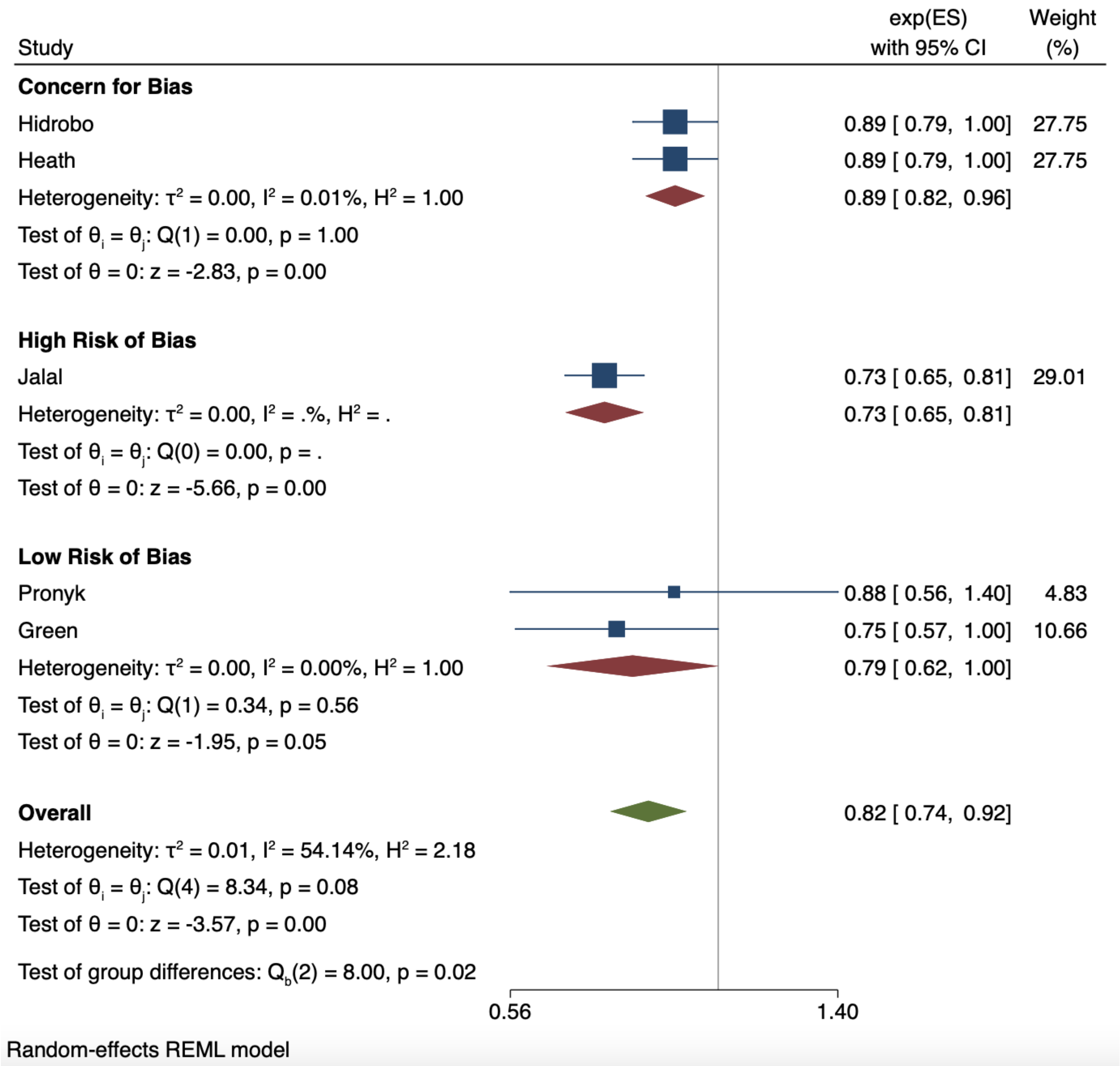

© 2023 Allan-Blitz LT et al. *JAMA Network Open.*

eFigure 4b: Leave-One-Out Analyses

Leave-one-out analysis for the impact of microfinance interventions on overall intimate partner violence

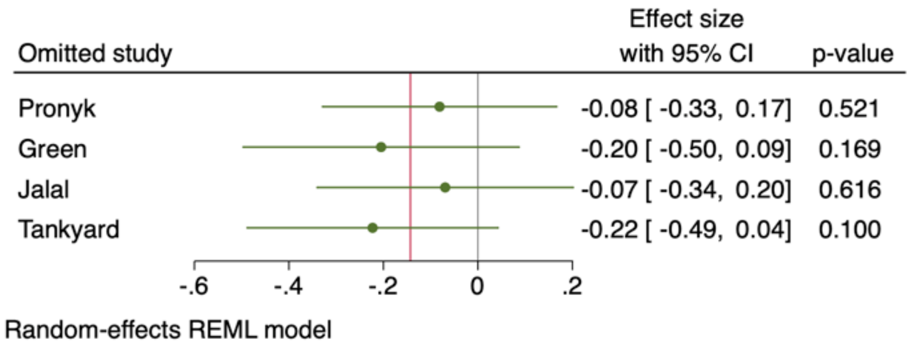

Leave-one-out analysis for the impact of microfinance interventions on physical intimate partner violence

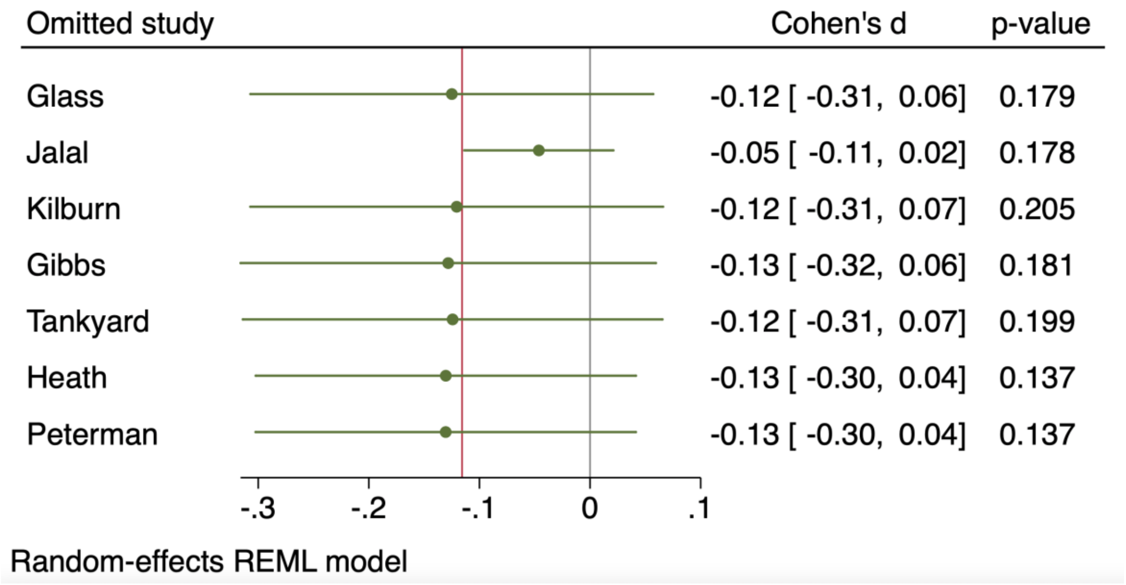

© 2023 Allan-Blitz LT et al. *JAMA Network Open.*

Leave-one-out analysis for the impact of microfinance interventions on psychological and emotional intimate partner violence

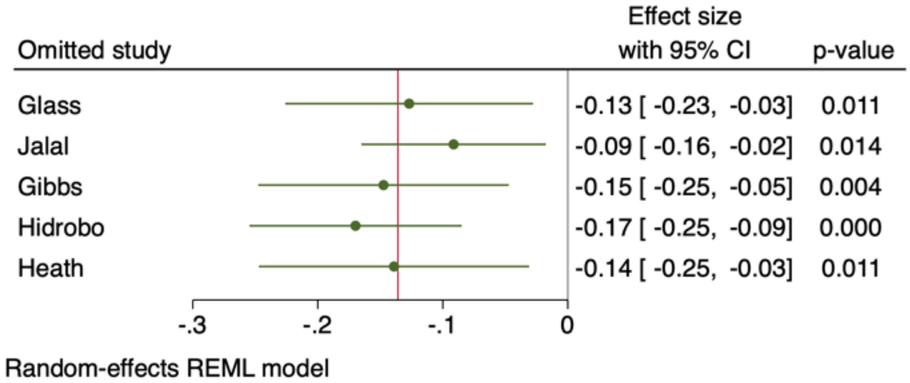

Leave-one-out analysis for the impact of microfinance interventions on controlling behaviors

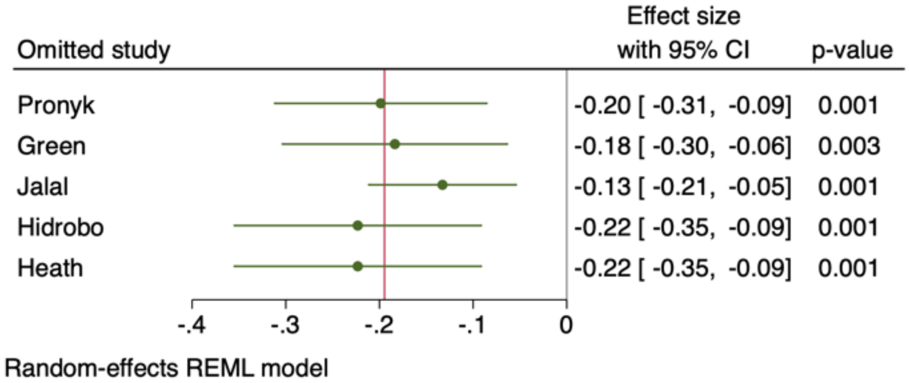

© 2023 Allan-Blitz LT et al. *JAMA Network Open.*

eFigure 5. Galbraith Plots and Funnel Plots Assessing for Heterogeneity and Publication Bias

Overall Intimate Partner Violence

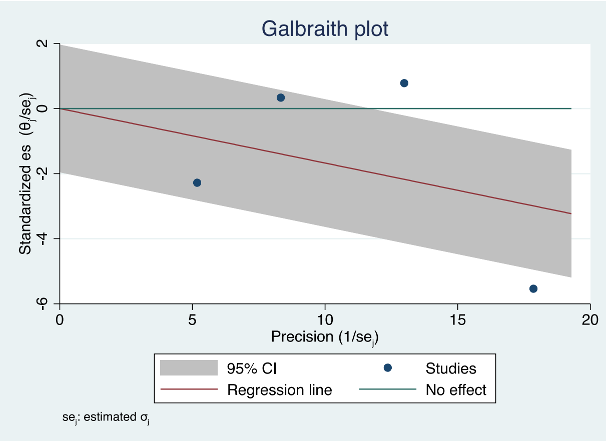

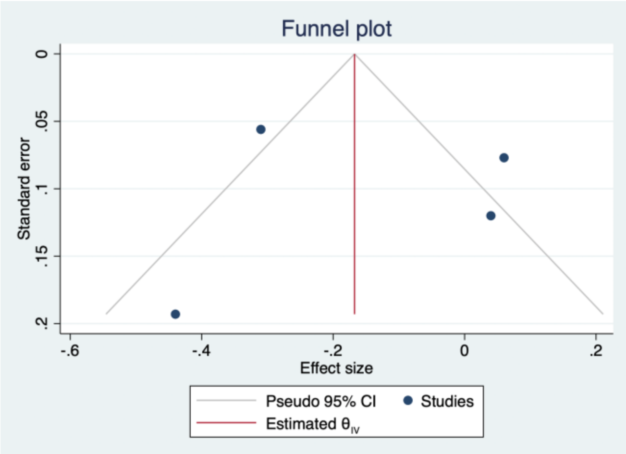

Physical Intimate Partner Violence

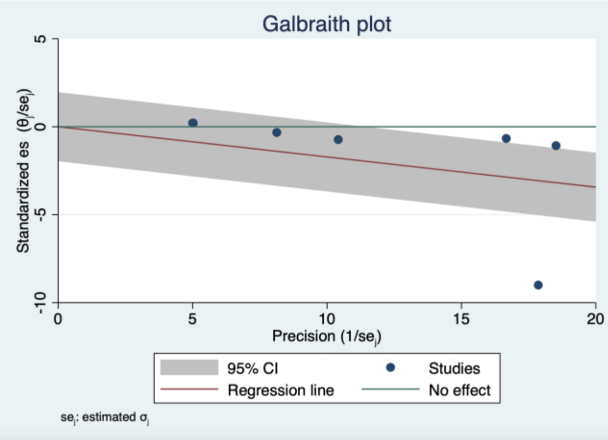

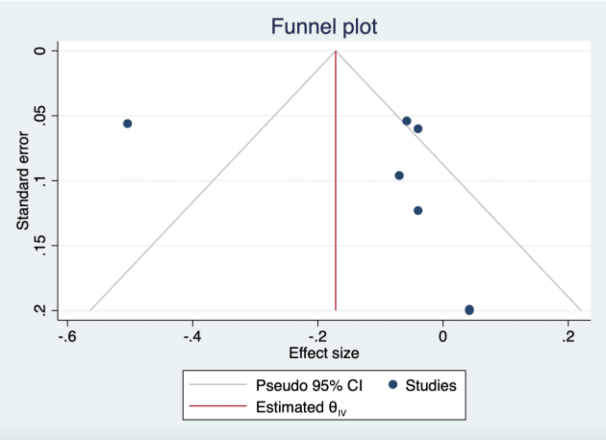

© 2023 Allan-Blitz LT et al. *JAMA Network Open.*

Psychological and Emotional Intimate Partner Violence

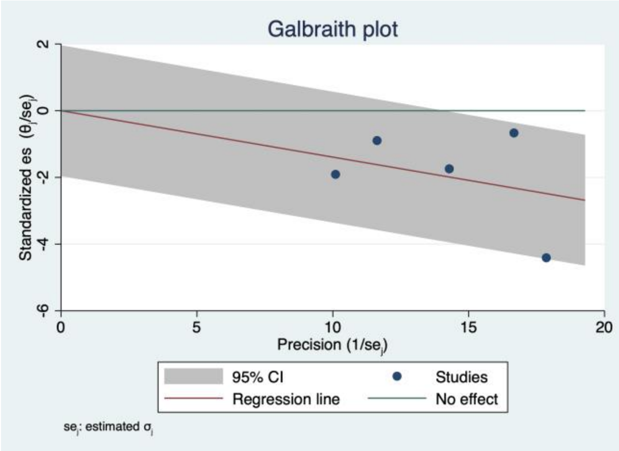

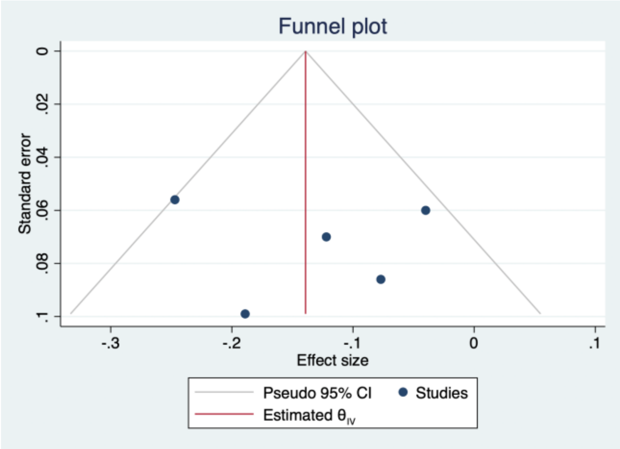

Sexual Intimate Partner Violence

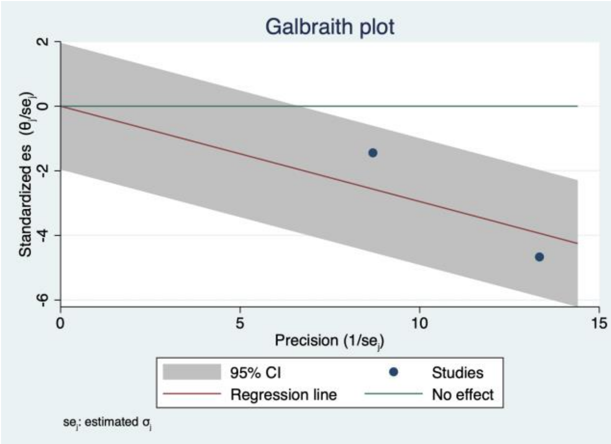

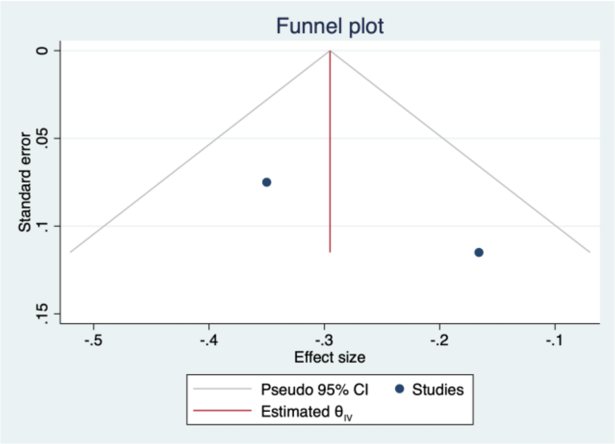

Controlling Behaviors

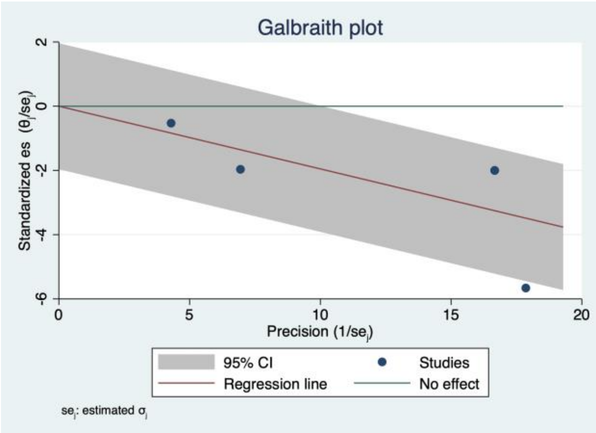

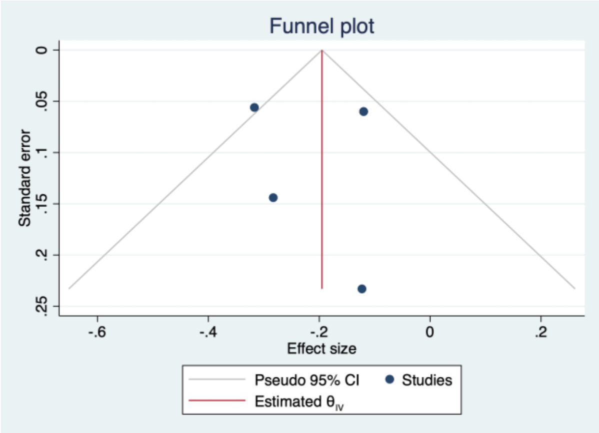

© 2023 Allan-Blitz LT et al. *JAMA Network Open.*
