# Supplementary material for: Assessment of Microfinance Interventions and Intimate Partner Violence: A Systematic Review and Meta-analysis
Source: JAMA Netw Open. 2023 Jan 27;6(1):e2253552. doi: 10.1001/jamanetworkopen.2022.53552 (PMC12543409; doi:10.1001/jamanetworkopen.2022.53552)
Supplement: Supplement 2. — Data Sharing Statement [file jamanetwopen-e2253552-s002.pdf]

Data Sharing Statement

Allan-Blitz. Assessment of Microfinance Interventions and Intimate Partner Violence. *JAMA Netw Open*. Published January 27, 2023. doi:10.1001/jamanetworkopen.2022.53552

Data
Data available: No

Additional Information
Explanation for why data not available: As the data are from already published reports, and no new data have been generated, there seems little value in publishing the data for this study. However, we are happy to share our data upon request.
